# Novel Use of an Image-Guided Stereotactic Approach in Trauma for Localization of Transcranial Bullet

**DOI:** 10.7759/cureus.1501

**Published:** 2017-07-21

**Authors:** Dat T Vo, George F Cravens, Robert E Germann

**Affiliations:** 1 Radiation Oncology, UT Southwestern; 2 Neurosurgery, John Peter Smith Hospital

**Keywords:** gunshot wound, penetrating brain injury, image-guided surgery, stereotactic neurosurgery, computed tomography

## Abstract

Penetrating brain injuries from gunshot wounds can carry a poor prognosis and require an aggressive, multifaceted approach to obtain a good prognosis and outcome. An initial evaluation requires appropriate imaging studies followed by management and prophylaxis against increased intracranial pressure, infection, and seizures. Surgical management is then followed to ensure the watertight closure of any wounds, removal of any areas of hematoma, and removal of any potential areas of infection. In this paper, we report the case of a patient who presented with a self-inflicted gunshot wound to the head and then received aggressive medical and surgical management. This case presents that an image-guided stereotactic approach with suitable medical management should be used in patients with penetrating missile injuries to the head.

## Introduction

Gunshot wounds to the brain present a very emergent situation requiring an aggressive approach. Many of these patients have inimitable socioeconomic situations that require special attention while managing the injury. A basic trauma assessment of the patient is required to allow for the comprehensive assessment of the patient and their presenting injuries. To assess the degree of injury, computed tomography (CT) is an appropriate first step. If a vascular injury is suspected, cerebral angiography can be proposed as well as intracranial pressure monitoring if appreciable cerebral edema is suspected. With many civilian firearms, the low-velocity nature of the missile would be retained in the brain tissue, which can provide a nidus for infection; this would require aggressive antibiotic prophylaxis to prevent it. In addition, the incidence of post-traumatic epilepsy is high in patients with penetrating brain injuries, and these patients would benefit from antiseizure prophylaxis.

Stereotactic neurosurgery allows for the localization of a surgical area of interest, especially in the eloquent and deep areas of the brain in three-dimensional space. In a frame-based system, a rigid stereotactic frame is placed on the patient under local anesthesia. Then, a CT or magnetic resonance imaging (MRI) scan of the brain is obtained with the frame on the patient. A localizing fiducial box is placed on the frame during the time of image acquisition. The target of interest is then localized using the Cartesian coordinates, x, y, and z, following image registration. The position of the target is now known, which is relative to the location of the frame in three-dimensional space, allowing for the precise targeting of the area of interest during surgery.

Here, we present a case of a patient who presented with a self-inflicted gunshot wound. The patient was aggressively managed with antibiotic and anti-seizure medications. The patient was then surgically managed for the entrance wound, requiring an image-guided stereotactic approach for debridement for the removal of the bullet fragment on the contralateral portion of the skull.

## Case presentation

Preoperative Information

A 55-year-old female presented to the hospital after transport via ambulance with a gunshot wound to the right temple. No loss of consciousness was noted, and the patient complained of a headache on the right. The patient had one episode of emesis that was relieved with medication. Prior to arrival, the patient was placed in a cervical spine collar. The patient had a Glasgow coma scale (GCS) of 14, presenting with confusion and disorientation. In the trauma bay, a central venous catheter was placed in the left femoral vein since the neck was not able to be accessed due to the cervical collar and the anxiety of the patient. Because of the penetrating injury to the head, the patient was placed on ceftriaxone, mannitol, and fosphenytoin and was taken to the intensive care unit (ICU) for further care.

Laboratory and Imaging Studies

**Figure 1:**
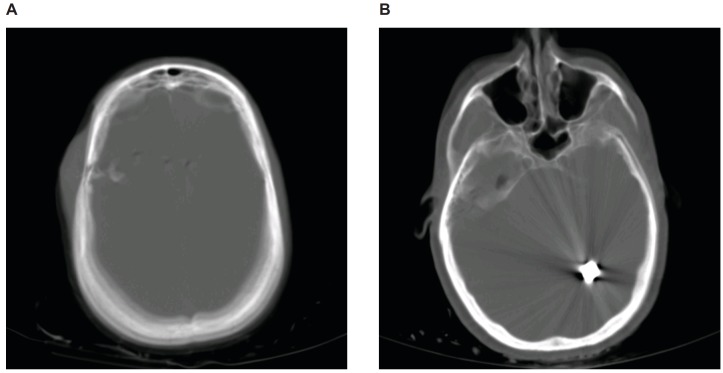
CT study of the head of the gunshot wound on admission (A) A comminuted fracture of the right parietal skull with displacement of the bony fragments is visualized. There is also a 2.2 centimeter displacement of the fragments into the right frontal temporal region. Soft tissue trauma with subcutaneous hematoma is also visualized over the lateral right parietal scalp. (B) A large bullet fragment is seen in the left parietal occipital region. Both panels are shown in the bone window.

Laboratory tests were largely unremarkable except for a blood alcohol level of 151 mg/dL and a positive drug screen for benzodiazepines. A CT study of the head showed a depressed and comminuted right skull fracture and a large, metallic bullet fragment in the left parietal occipital region, consistent with a self-inflicted gunshot through the lateral right temporal skull, directed posterior medially(Figure 1).

A 4 millimeter right-to-left midline shift was also noted. A CT study of the abdomen and pelvis did not show any acute traumatic injuries. Two subsequent CT studies of the head showed a mildly increasing edema and ischemia on the right portion of the bullet track. On imaging, no evidence suggested any vascular injuries requiring cerebral angiography.

Operative Intervention and Surgery

The patient underwent surgery on hospital Day 4 with a right temporal craniectomy with the incision and drainage of the gunshot entrance wound and an image-guided left occipital craniotomy with the removal of the bullet. Due to the location of the bullet and injury, a stereotactic approach was used. Prior to surgery, the patient had a VectorVision (BrainLab, Inc., Westchester, IL) CT scan. The Stryker image-guided stereotactic system (Stryker, Kalamazoo, MI) was used following anatomical registration for locating the trajectory and location of the bullet. A craniectomy was performed on the right temporal area, where bullet fragments, bone fragments, hair, and soft tissue were removed. The area was irrigated and then covered with Duragen (Integra, Plainsboro, NJ). The temporalis muscle was then reapproximated with sutures, and the skin was stapled.

The focus was then placed on the left occipital region with the use of the stereotactic system to determine the trajectory and depth of the bullet. An incision was then made in the left occipital area with a self-retaining retractor placed. A small craniectomy was performed on the occipital bone. Using the stereotactic system, the trajectory and depth of the bullet were determined. A small cerebrotomy was performed, and a dissection was carried down to the bullet fragment. A large jacketed bullet, approximately 32 caliber, was removed. The area was copiously irrigated, and the dura was reapproximated. The dura was covered with Duragen. The bone was replaced using the Aesculap cranial fixation system (Aesculap, Inc., Center Valley, PA). The galea was reapproximated using sutures, and the skin was stapled. Somatosensory evoked potentials (SEP) and electroencephalography showed baseline activities.

Surgical Pathology 

The right temporal bone debridement showed viable bone fragments with medullary hemorrhage and acute inflammation.

Postoperative Course 

**Figure 2:**
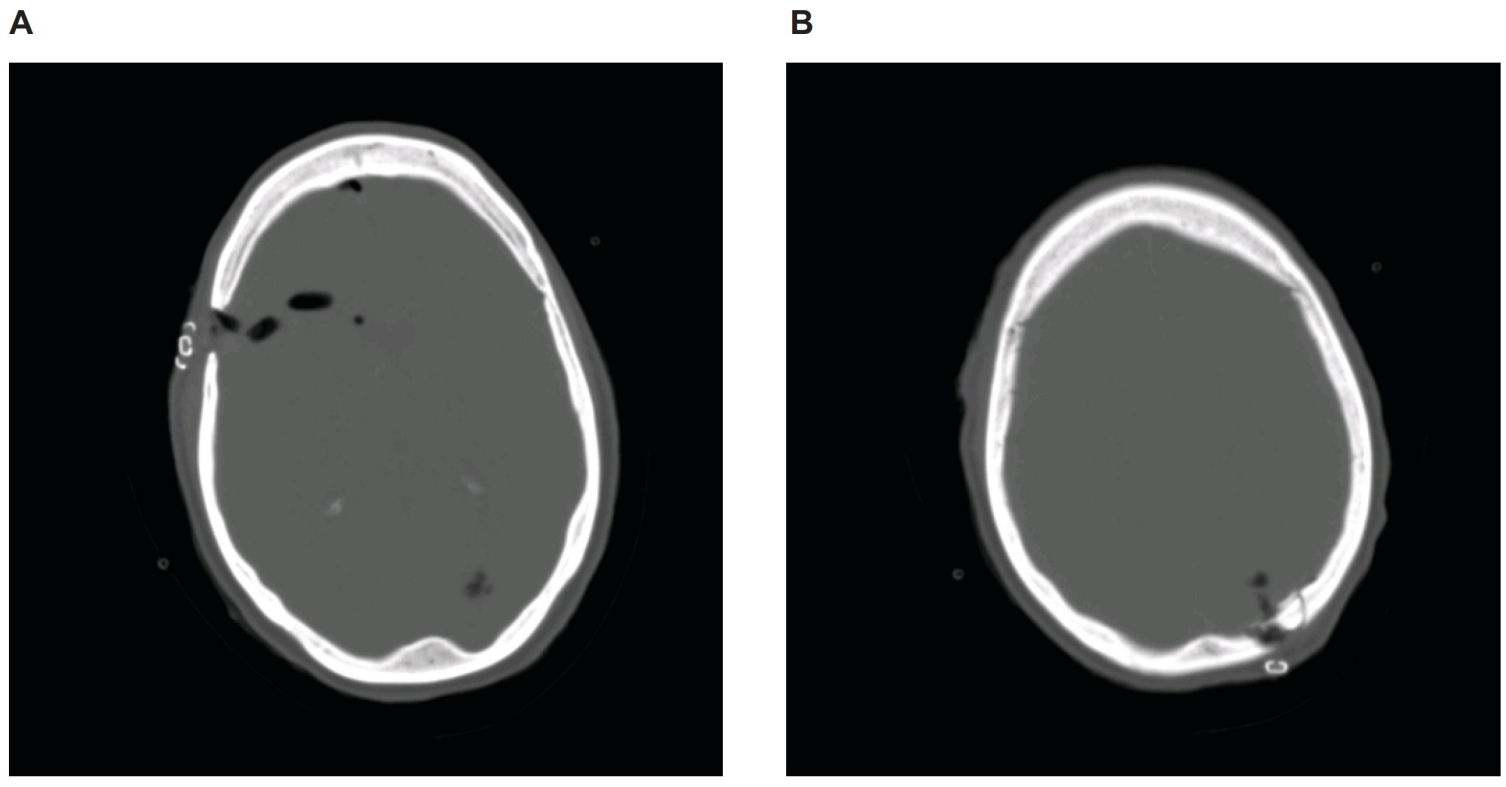
CT study of the head of the gunshot wound on postoperative Day 1 (A) Image shows the removal of bone fragments in the right frontal region with mild pneumocephalus. (B) Image shows resection of the bullet fragment in the left parietal region. Both panels are shown in the bone window.

The patient recovered extremely well after surgery, with good follow-up imaging studies (Figure 2).

The patient complained of pain and headache, which was controlled with medication. Concerns arose regarding dysphagia, but the swallowing function was regained after a couple of days. The patient was evaluated by psychiatry who deemed that the injury was due to a suicide attempt. The patient was transferred to inpatient rehabilitation for further psychiatric care and treatment.

## Discussion

Since the first description of the use of stereotaxy in neurosurgery [1], image-guided surgery has become an essential tool in the neurosurgeon’s tool box. While initial studies utilized anatomical landmarks for delineating intracranial coordinates, modern techniques utilize CT and MRI scans to visualize the surgical and target site more precisely and at high resolution. For patients with a traumatic, penetrating brain injury from a gunshot wound, stereotactic methods are a useful tool for removing the missile and for healing the associated injuries with favorable outcomes [2-4]. Stereotactic neurosurgery is an ideal approach for our patient as it allows for the removal of the bullet, with as minimal damage to the adjacent normal tissue as possible, allowing for an increased chance of functional recovery.

Our patient presented with a gunshot wound through the right temporal region with the bullet finally residing in the left parietal occipital region, in a presumed suicide attempt. Considering the close range of the gun, the bullet carried the same velocity when it entered the brain as it did when it exited the gun, without any effect of air resistance on the bullet itself. Since the velocity of the bullet predominates in determining the kinetic energy of any given projectile mass (Equation 1), the bullet itself will cause significant soft tissue damage, creating a track of injury [5]. 

Equation 1: $E=\frac{1}{2}mv^{2}$

In addition, with high velocity and energy, a proportion of energy is transferred to the surrounding brain tissue, causing compression and re-expansion with a resultant compressive tissue damage [6].

We report on a patient with a self-inflicted gunshot wound that penetrated the right temporal skull with the resultant location of the projectile being the left parietal occipital region of the brain. In many civilian gunshots, the location of the projectile is also associated with death if there is acute pressure on the brainstem [7]. Our patient was surgically managed using an image-guided approach for the adequate debridement of the wound and injury [8]. Our patient had a number of factors (normotensive blood pressure, respiratory status, GCS, pupillary size and reflex, and imaging findings) that portended a good prognosis [9]. Our patient also received aggressive anti-seizure and antibiotic prophylaxis to ensure a good outcome and a reduced amount of complications.

## Conclusions

To this end, we report on a patient with a self-inflicted gunshot wound to the head that required a unique image-guided stereotactic surgical approach. The patient was treated with a surgical approach coupled with aggressive antibiotic and anti-seizure prophylaxis, following the guidelines for the management of penetrating brain injuries. We propose an aggressive, multifaceted approach to obtain a good outcome in patients with penetrating brain injuries.
